# CT Scan Does Not Differentiate Patients with Hepatopulmonary Syndrome from Other Patients with Liver Disease

**DOI:** 10.1371/journal.pone.0158637

**Published:** 2016-07-06

**Authors:** Yingming Amy Chen, Vikramaditya Prabhudesai, Helene Castel, Samir Gupta

**Affiliations:** 1 Department of Medical Imaging, St. Michael’s Hospital, University of Toronto, Toronto, Canada; 2 Département de Medecine, Université de Montréal, Montreal, Canada; 3 Division of Respirology, Department of Medicine, St. Michael’s Hospital, University of Toronto, Toronto, Canada; 4 Li Ka Shing Knowledge Institute, St. Michael’s Hospital, Toronto, Canada; University of Louisville, UNITED STATES

## Abstract

**Background:**

Hepatopulmonary syndrome (HPS) is defined by liver dysfunction, intrapulmonary vascular dilatations, and impaired oxygenation. The gold standard for detection of intrapulmonary vascular dilatations in HPS is contrast echocardiography. However, two small studies have suggested that patients with HPS have larger segmental pulmonary arterial diameters than both normal subjects and normoxemic subjects with cirrhosis, when measured by CT. We sought to compare CT imaging-based pulmonary vasodilatation in patients with HPS, patients with liver dysfunction without HPS, and matching controls on CT imaging.

**Methods:**

We performed a retrospective cohort study at two quaternary care Canadian HPS centers. We analyzed CT thorax scans in 23 patients with HPS, 29 patients with liver dysfunction without HPS, and 52 gender- and age-matched controls. We measured the artery-bronchus ratios (ABRs) in upper and lower lung zones, calculated the “delta ABR” by subtracting the upper from the lower ABR, compared these measurements between groups, and correlated them with clinically relevant parameters (partial pressure of arterial oxygen, alveolar-arterial oxygen gradient, macroaggregated albumin shunt fraction, and diffusion capacity). We repeated measurements in patients with post-transplant CTs.

**Results:**

Patients had significantly larger lower zone ABRs and delta ABRs than controls (1.20 +/- 0.19 versus 0.98 +/- 0.10, p<0.01; and 0.12 +/- 0.17 versus -0.06 +/- 0.10, p<0.01, respectively). However, there were no significant differences between liver disease patients with and without HPS, nor any significant correlations between CT measurements and clinically relevant parameters. There were no significant changes in ABRs after liver transplantation (14 patients).

**Conclusions:**

Basilar segmental artery-bronchus ratios are larger in patients with liver disease than in normal controls, but this vasodilatation is no more severe in patients with HPS. CT does not distinguish patients with HPS from those with uncomplicated liver disease.

## Introduction

Hepatopulmonary syndrome (HPS) is a rare pulmonary vascular complication of liver disease, defined by hepatic dysfunction, intrapulmonary vascular dilatations (IPVDs), and impaired oxygenation. HPS affects 8–33% of patients with liver disease, is rapidly progressive[1], and more than doubles the hazard of death compared to liver disease alone[2].

IPVDs are thought to be caused by nitric oxide-mediated vasodilatation of muscularized arterioles immediately proximal to the pulmonary capillaries, resulting in flow-mediated dilatation of alveolar septal capillaries[3]. In severe cases, this leads to impairment of oxygenation, resulting in HPS. The gold standard for detection of these microscopic IPVDs is contrast echocardiography, with delayed passage of microbubbles into the left atrium indicating passage through dilated pulmonary vessels[1].

Previous authors have noted that chest radiographs may show basilar reticulonodular opacities in HPS, which correspond to dilated pulmonary vessels on conventional CT[4]. Two prior studies have suggested that patients with HPS have larger segmental pulmonary arterial diameters than both normal subjects and normoxemic subjects with cirrhosis, when measured by high resolution CT[5,6]. However, both were small studies subject to methodological limitations.

We sought to characterize and compare CT imaging-based pulmonary vascular abnormalities in larger cohorts of patients with and without HPS, to compare these groups to normal controls, and to correlate their CT findings with clinically relevant parameters.

## Materials and Methods

### Study Population

We retrospectively analyzed consecutive patients seen at two specialized quaternary care HPS clinics, from February 2001 (clinic 1 –Hôpital St-Luc, Montreal, Canada), and June 2004 (clinic 2 –St. Michael’s Hospital, Toronto, Canada) to October 2012. Institutional review board approvals were received at each institution (Le comité d’éthique de la recherche du Centre Hospitalier de l'Universite de Montreal (CHUM) 07.092, St. Michael's Hospital (SMH) Research Ethics Board 10-155/University Health Network Research Ethics Board 12–0280) and patient information was anonymized and de-identified prior to analysis.

We included all subjects who had an available CT scan and had either HPS [with imaging and/or clinical evidence of liver dysfunction and/or portal hypertension, contrast echocardiographic evidence of IPVDs (microbubbles in the left atrium ≥3 cardiac cycles after appearance in the right atrium) and arterial blood gas evidence of oxygenation abnormality (partial pressure of arterial oxygen (PaO_2_) ≤70 mmHg and alveolar-arterial oxygen gradient (AaDO_2_) >20 mmHg on room air, as described previously][6–8]; or liver dysfunction without HPS [liver dysfunction and/or portal hypertension but no significant oxygenation abnormality]. We excluded subjects with non-diagnostic echocardiograms, pulmonary hypertension (echocardiographic estimated right ventricular systolic pressure ≥50 mmHg and/or right heart catheterization mean pulmonary artery pressure >25 mmHg with pulmonary capillary wedge pressure ≤15 mmHg), a baseline FEV1/FVC ratio <0.65 or TLC <70% predicted[9], and those with a known diagnosis of interstitial lung disease (ILD), bronchiectasis, moderate/severe chronic obstructive pulmonary disease (COPD) or asthma[10], or moderate/large pleural effusion(s). Upon CT scan analysis, we further excluded subjects whose CT showed evidence of bronchiectasis [defined as pulmonary artery-bronchus ratio (ABR) >1.5], any parenchymal disease, moderate/large pleural effusion(s) (defined as effusion large enough to cause passive or compressive atelectasis), or motion artefact precluding accurate bronchovascular measurements.

In cases where more than one CT was available, we analysed the CT closest in date to the contrast echocardiogram. For each included subject, we identified a gender and age-matched (within 3 years) control subject with a CT pulmonary angiogram that had been interpreted as within normal limits (performed between 2008 and 2012, through the emergency department). We also analyzed post-transplant CT scans in any included patients who subsequently received liver transplantation (CT performed ≥3 months post-liver transplant and PaO_2_ improved ≥10 mmHg in HPS patients). In cases where more than one post-transplant CT was available, we analysed the CT closest to one year post-transplant, by which time a full reversal of HPS would be expected[11].

### CT Measurements

**Fig 1:**
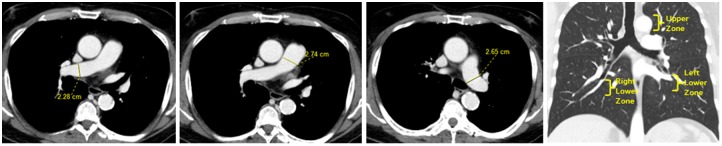
CT scans from a control subject demonstrating bronchovascular measurements. Axial images demonstrate measurements of the main pulmonary artery (A), the right pulmonary artery (B), and the left pulmonary artery (C). Coronal image from the same subject demonstrates location of the upper and lower zones for bronchovascular measurements (brackets) (D).

**Fig 2:**
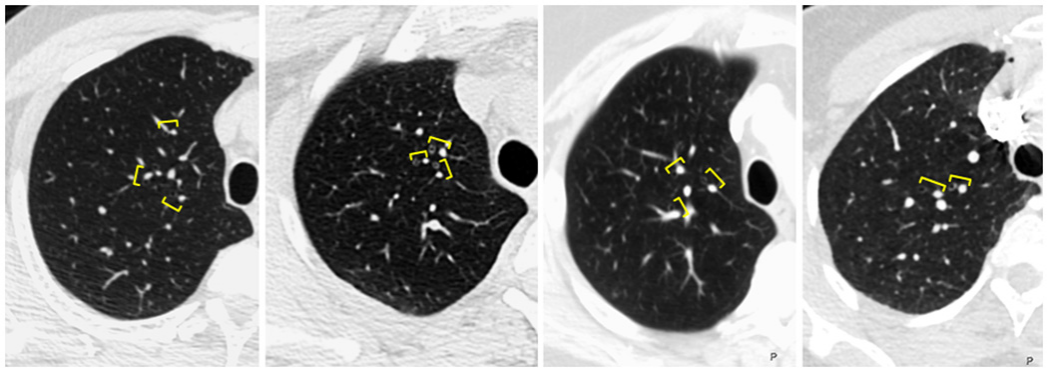
Axial CT images of the right upper zone in a patient with hepatopulmonary syndrome (A), subclinical hepatopulmonary syndrome (B), liver dysfunction only (C), and control subject (D) with representative artery and accompanying bronchus highlighted (brackets).

**Fig 3:**
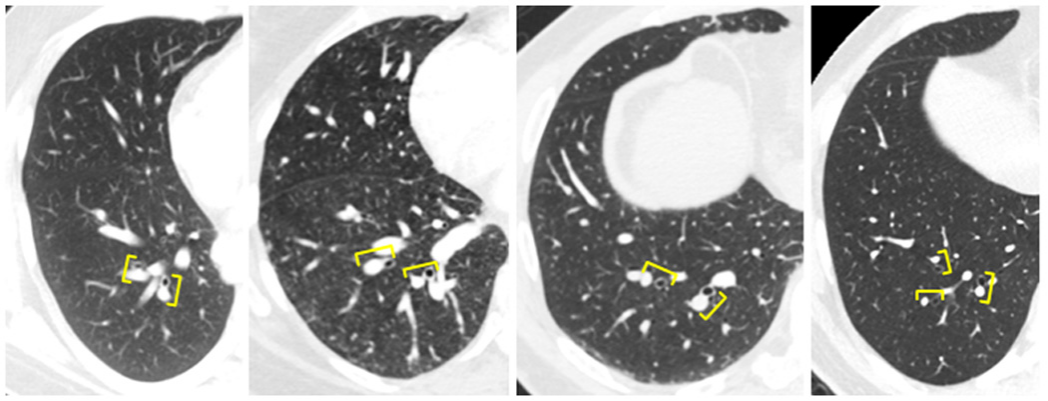
Axial CT images of the right lower zone in a patient with hepatopulmonary syndrome (A), subclinical hepatopulmonary syndrome (B), liver dysfunction only (C), and control subject (D) with representative artery and accompanying bronchus highlighted (brackets).

All measurements were performed by an investigator who was blinded to patient/control status. We measured pulmonary vascular diameters manually on 1-mm slice axial reconstructions, using the digital caliper tool from a commercially available image-processing software (TeraRecon Aquarius iNtuition, Foster City, CA). We measured diameters of the central pulmonary arteries on mediastinal windows (400HU), just above the pulmonic valve. The widest diameter perpendicular to the long axis of the main pulmonary artery (MPA) was measured at the level of the bifurcation of the pulmonary artery, and diameters of the right and left pulmonary arteries were measured at their widest portion before branching (Fig 1). We measured diameters of segmental pulmonary arteries and bronchi on lung windows (1600 HU). In order to account for the known basilar predominance of IPVDs in HPS[12,13], we divided the bronchovasculature into upper and lower zones in each lung and measured the transverse diameters of the three largest segmental pulmonary arteries and their accompanying airways in each zone. Upper zone measurements were made within the contiguous slices spanning the height of the aortic arch and lower zone measurements were made in the 20 contiguous slices (2 cm) distal to the take-off of the basal segmental arteries (Fig 1). We calculated the ABR as the ratio of the diameter of the segmental pulmonary artery to the outer diameter of its accompanying bronchus. This measurement was preferred to vessel size alone because it adjusts for anatomic variables such as lung size, individual breath size, age-related effects on bronchovascular diameter[14], and variability introduced by measurements made at different levels of the segmental bronchovasculature[15]. We measured three ABRs in each of the upper (Fig 2) and lower (Fig 3) zones of each lung, and calculated “delta ABR” by subtracting the mean upper ABR from the mean lower ABR.

### Clinical Parameters

All patients had clinical exam, blood work and pulmonary function testing, including diffusion capacity (DLCO)[16]. Patients with HPS had macroaggregated albumin (MAA) shunt testing, analyzed through the Abrams technique[17]. All patients had an arterial blood gas on room air, at rest in the seated or standing position[18,19]. In patients with more than one test result, we used results closest to the date of the CT scan.

### Statistical Analysis

Data are expressed as proportions (percentages), means and standard deviations. All continuous variables were tested for normality. Continuous variables were compared with a 2-sample t-test (pairwise comparisons) and an ANOVA (three group comparisons), and categorical variables with a Chi-squared test or Fisher’s exact test, as appropriate. The primary outcome was the difference in delta ABR between groups. We used paired t-tests to compare upper and lower ABRs in patients within each group, and to compare pre- and post-transplant values within patients. We used Pearson’s correlation coefficient to measure baseline correlations between CT measurements and clinical parameters (PaO_2_, AaDO_2_, MAA, or DLCO) and to measure correlations between changes in CT measurements and clinical parameters from pre- to post-transplant. P-values < 0.05 were considered statistically significant. All data were analyzed using SAS 9.4 for Windows (SAS Institute, Cary, NC).

## Results

### Study Population

113 patients met inclusion criteria. 57 were excluded before CT analysis for the following reasons: non-diagnostic echocardiogram (4); pulmonary hypertension (7); FEV1/FVC ratio <0.65 (18); TLC <70% predicted (7); and known ILD (5), bronchiectasis (3), moderate/severe COPD (9) or asthma (1), or moderate/large pleural effusion(s) (3). Upon CT scan analysis, an additional 4 patients were excluded due to: bronchiectasis (1), parenchymal consolidation (1), and image motion (2). We analyzed the remaining 52 patients, including 23 with HPS (44.2%) and 29 with liver dysfunction without HPS (55.8%). Patient characteristics are described in Table 1.

**Table 1:** Patient Demographic and Clinical Characteristics.

	Hepatopulmonary Syndrome (n = 23)	Liver Dysfunction Without Hepatopulmonary Syndrome (n = 29)	p-Value
Age (years)	54.0 +/-11.6	57.3 +/- 8.7	0.23
Female sex (%)	8 (34.8%)	6 (20.7%)	0.35
Liver disease Etiology (%)	Alcoholic = 8 (34.8%)	Alcoholic = 8 (27.6%)	0.04
	NASH = 5 (21.7%)	NASH = 2 (6.9%)	
	Hep C = 1 (4.3%)	Hep C = 11 (37.9%)	
	Hep C + alcoholic = 3 (13.0%)	Hep C + alcohol = 3 (10.3%)	
	Other = 6 (26.0%)	Other = 5 (17.2%)	
Childs-Pugh Class Distribution	A = 7 (30.4%) B = 14 (60.9%) C = 2 (8.7%)	A = 10 (34.5%) B = 17 (58.6%) C = 2 (6.9%)	1.0
MELD Score	12.7 +/- 3.7	12.8 +/- 4.4	0.91
Pulmonary Function			
FEV/FVC (%)	74.6 +/- 5.6	76.7 +/- 4.8	0.16
TLC (% pred)	96.0 +/- 18.0	98.3 +/- 18.9	0.68
DLCO (% pred)	50.2 +/- 13.4	79.5 +/- 12.4	<0.01
Oxygenation			
PaO_2_ (mm Hg)	52.2 +/- 10.4	91.7 +/- 10.3	<0.01
AaDO_2_ (mm Hg)	60.9 +/- 14.2	15.5 +/- 9.7	<0.01

Mean values are provided with standard deviations

Hep C denotes hepatitis C; MELD denotes Model for End-Stage Liver Disease; NASH denotes non-alcoholic steatohepatitis; pred denotes predicted

### CT Measurements (Table 2 Figs 2 and 3)

**Table 2:** Patient and Control CT Measurements.

	Hepatopulmonary Syndrome (n = 23)	Liver Dysfunction Without Hepatopulmonary Syndrome (n = 29)	Disease Group Comparisons (p-Value)	All Disease Patients (n = 52)	Controls (n = 52)	Disease-Control Comparisons (p-Value)
MPA (cm)	2.62 +/- 0.35	2.63 +/- 0.35	0.89	2.63 +/- 0.34	2.67 +/- 0.45	0.56
RPA (cm)	2.28 +/- 0.36	2.22 +/- 0.35	0.51	2.25 +/- 0.35	2.10 +/- 0.34	0.03
LPA (cm)	2.15 +/- 0.33	2.16 +/- 0.24	0.90	2.16 +/-0.28	2.09 +/- 0.29	0.23
Upper ABR	1.08 +/- 0.13	1.07 +/- 0.11	0.75	1.07 +/- 0.12	1.03 +/- 0.11	0.06
Lower ABR	1.16 +/- 0.18	1.22 +/- 0.20	0.27	1.20 +/- 0.19	0.98 +/- 0.10	<0.01
Delta ABR*	0.08 +/- 0.16	0.15 +/- 0.17	0.14	0.12 +/- 0.17	-0.06 +/- 0.10	<0.01

Mean values are provided with standard deviations

MPA denotes main pulmonary artery; RPA denotes right pulmonary artery; LPA denotes left pulmonary artery; ABR denotes artery-bronchus ratio

*Delta ABR was calculated by subtracting the upper ABR from the lower ABR

There were no significant differences in any of the CT measurements between liver disease patients with and without HPS. Patients with any disease had larger mean values for RPA, lower zone ABR, and delta ABR than control patients (Table 2). Similarly, in pairwise comparisons between each disease group and their matching controls, diseased patients in each group had significantly larger values for lower zone ABR and delta ABR than control patients (S1 Table). Within patients, lower ABRs were significantly larger than upper ABRs in patients with HPS (p = 0.02) and liver dysfunction without HPS (p<0.01), but significantly smaller in controls (p<0.01) (see ABR values in Table 2). We analyzed post-transplant CT scans in 14 patients (6 HPS, 8 liver dysfunction without HPS), and did not find significant changes in CT measurements after transplant in all patients, nor in HPS patients alone (S2 Table). We also further divided patients with liver dysfunction without HPS into those with “subclinical” HPS [liver dysfunction and/or portal hypertension, contrast echocardiographic evidence of IPVDs, but no significant oxygenation abnormality (AaDO2≤20mmHg, and PaO2>70mmHg)] (22 patients) and those with liver dysfunction only (liver dysfunction and/or portal hypertension with negative contrast echocardiography) (7 patients). There remained no significant differences in any of the CT measurements between groups (S3 Table).

### Clinical Parameters

There were no significant correlations between any baseline CT measurements nor between pre- to post-transplant changes in any CT measurements and any of: PaO_2_, AaDO_2_, MAA, or DLCO (tested in all patients and in HPS patients alone).

## Discussion

We analyzed artery-bronchus ratios (ABRs) in carefully phenotyped disease cohorts, and found that both patients with HPS and those with liver dysfunction without HPS had dilated basal segmental pulmonary arteries compared to controls, but found no inter-group differences. This suggests that CT scan findings do not make the important clinical distinction between patients with and without HPS.

Our finding that basal segmental ABRs are larger in patients with liver disease (including those with or without IPVDs and hypoxemia) than in normal controls was also previously reported by Koksal, et al.[5]. This finding is congruent with an extensive literature examining the hyperdynamic circulatory state of cirrhosis, believed to be mediated by a host of vasoactive substances that cause diffuse vasodilatation of muscularized arteries including pulmonary arterioles[20,21]. Since these mediators enter the pulmonary vascular bed directly through the pulmonary artery and pulmonary arterial flow is maximally distributed to basilar vessels due to gravity[12], it follows that lower zone ABRs were affected more so than upper zone ABRs.

However, we did not find a gradient in segmental ABR size between patients with HPS, subclinical HPS, and liver dysfunction alone. This can be explained by the fact that although segmental (proximal muscularized) pulmonary artery vasodilates in patients with cirrhosis, IPVDs constitute a distinct anatomic abnormality located distal to segmental vessels[5,6]. Pathologic studies have defined IPVDs as dilatations of alveolar septal arterioles and capillaries, from a normal diameter of 7–15 uM[22] to 60–80 uM[3,13]. The pathophysiologic mechanism of these IPVDs is also distinct from the generalized vasodilatation of cirrhosis, and thought to be related to frank vascular remodelling and angiogenesis at the pre-capillary level, rather than a simple loss of vascular tone[1]. These dilatations are diagnosed indirectly by the transpulmonary passage of either saline microbubbles (measuring 35–90 uM)[3,13,23] in contrast echocardiography, or albumin macroaggregates (measuring 20–80 uM)[3,12,23] in the MAA nuclear shunt test. They are likely below the minimum spatial resolution of HRCT, which ranges from 100–500 uM, depending on scanner type and protocol[24,25]. This also explains the observed lack of correlation between CT measurements and MAA shunt results.

Two previous studies have analysed CT findings in HPS[5,6] and found higher basilar ABRs in HPS compared to non-hypoxemic cirrhotics; however, both studies were much smaller than ours and had methodologic limitations. Lee, et al. performed an unblinded comparison of ABRs in 4 pairs of patients with HPS versus nonhypoxemic cirrhosis (IPVD status unknown), and measured 12 ABRs in each patient, treating these as individual data points without adjusting for clustering effects. Koksal, et al. compared 10 patients with HPS to 12 patients with liver dysfunction only, and did not include patients with subclinical HPS. Notably, a single HPS patient in Koksal’s study had a basilar arterial diameter of 9 mm, which was 1.5 times the next largest value and nearly twice the group mean[5]. This data point not only skewed the ABR analysis, but a segmental vessel of this caliber more likely represented the feeding artery of a pulmonary arteriovenous malformation, which is a radiographically distinct entity reported in HPS[26]. Furthermore, neither of the studies reported exclusion criteria for patients with intrinsic lung disease, nor pulmonary function values of included patients, and neither sought age- or gender-matched controls.

Although the mean basilar ABR measured in our normal subjects (0.98 +/- 0.10) was similar to that reported elsewhere (0.98 +/- 0.14)[15], measurement techniques were also different in our study than in these two previous studies. We chose to measure outer bronchial wall diameters instead of luminal diameters for ABR calculations[5,6] because they are less susceptible to the effects of bronchial wall thickening, secretions and bronchospasm[27–29], and more reliably measured than luminal diameters[30]. We defined the lower lung zone as the 20 contiguous slices after take-off of the basal segmental arteries, whereas both prior authors defined this more distally, as a region within 2 cm of the lung periphery[5,6]. However, other authors have noted that bronchi are not visible within the peripheral 2 cm of the lung on CT[24], and we were similarly unable to accurately identify bronchi within this region in our subjects. Furthermore, pulmonary arteries in the peripheral 2 cm of the lung are usually subsegmental and expected to be much smaller than the sizes reported in both papers (means of 3.7 mm[5] and 5.0 mm[6] in healthy controls).

To our knowledge, no previous study has assessed CT scan changes after transplantation in this population. Observed baseline increases in lower zone ABRs across these groups did not return to normal after transplant in our small sample of 14 patients. We analyzed CT scans performed a minimum of 3 months after transplant, and it is possible that this cut-off was insufficient for cirrhosis-related macrovascular changes to reverse[11]. It is also possible that some vascular abnormalities may persist indefinitely, as suggested by several previous studies which found persistent diffusion abnormalities in patients post-liver transplantation, despite improved gas-exchange[31–33].

Our study has several limitations. Minimum resolution varies according to acquisition protocol, and all control studies were CT pulmonary angiogram protocols, whereas patient studies had variable acquisition protocols. However, measurement variability due to variable scan resolutions between disease patients would not be expected to be directional in nature, and would likely affect upper zone measurements more so than those in lower zones (and there were no observed inter-group differences for either zone). Next, there is currently no gold standard for segmental artery or ABR measurement, and it is possible that a different measurement technique would be able to identify inter-group differences which our technique could not. However, we chose a measurement technique that is practically reproducible and applicable in most settings. More specialized techniques would not be generalizable to the typical practice environment, and therefore of limited clinical utility.

## Conclusions

In summary, we characterized pulmonary vascular abnormalities in a carefully phenotyped cohort of patients with liver disease, and found evidence of basilar pulmonary arterial dilatation on CT scans of patients with liver disease compared to control subjects, but no differences between liver disease patients with and without HPS. Our results suggest that the systemic vasodilatation of liver disease can be detected on CT scan, whereas the microscopic IPVDs which characterize HPS cannot be detected, and CT scan does not have a role in HPS diagnosis.

## Supporting Information

S1 Table Comparison of pulmonary bronchovascular measurements between disease groups and matched controls. (DOCX)

S2 Table Comparison of pulmonary bronchovascular measurements pre- vs. post-liver transplantation. (DOCX)

S3 Table Comparison of pulmonary bronchovascular measurements among subdivided liver disease groups. (DOCX)
