# Supplementary material for: CT Scan Does Not Differentiate Patients with Hepatopulmonary Syndrome from Other Patients with Liver Disease
Source: PLoS One. 2016 Jul 6;11(7):e0158637. doi: 10.1371/journal.pone.0158637 (PMC4934684; doi:10.1371/journal.pone.0158637)
Supplement: S1 Table — (DOCX) [file pone.0158637.s001.docx]

Supporting Information

**S1 Table. Comparison of pulmonary bronchovascular measurements between disease groups and matched controls.**

|  | HPS (n=23) | HPS Matched Controls (n=23) | HPS-Control Comparisons (p-value) | Liver Dysfunction Without HPS (n=29) | Liver Dysfunction Without HPS Matched Controls (n=29) | Liver Dysfunction Without HPS-Control Comparisons (p-value) |
| --- | --- | --- | --- | --- | --- | --- |
| Lower ABR | 1.16 +/- 0.17 | 0.99 +/- 0.091 | <0.0001 | 1.22 +/- 0.20 | 0.97 +/- 0.11 | 0.0001 |
| Delta ABR | 0.082 +/- 0.16 | -0.037 +/- 0.11 | 0.005 | 0.15 +/- 0.17 | -0.07 +/- 0.090 | <0.0001 |

Mean values are provided with standard deviations

HPS denotes hepatopulmonary syndrome, ABR denotes artery-bronchus ratio

*Delta ABR was calculated by subtracting the upper ABR from the lower ABR
