# Supplementary material for: CT Scan Does Not Differentiate Patients with Hepatopulmonary Syndrome from Other Patients with Liver Disease
Source: PLoS One. 2016 Jul 6;11(7):e0158637. doi: 10.1371/journal.pone.0158637 (PMC4934684; doi:10.1371/journal.pone.0158637)
Supplement: S2 Table — (DOCX) [file pone.0158637.s002.docx]

Supporting Information

**S2 Table. Comparison of pulmonary bronchovascular measurements pre- vs. post-liver transplantation.**

|  | HPS Pre- vs. Post-Transplant Difference (n=6) | HPS Pre- vs. Post-Transplant Comparisons (p-value) | All Liver Disease (HPS and Liver Dysfunction Without HPS) Pre- vs. Post-Transplant Difference (n=14) | All Liver Disease Pre-vs. Post-Transplant Comparisons (p-value) |
| --- | --- | --- | --- | --- |
| MPA (cm) | 0.017 +/- 0.87 | 0.96 | -0.21 +/- 0.66 | 0.25 |
| RPA (cm) | -0.33 +/- 0.53 | 0.88 | -0.14 +/- 0.57 | 0.36 |
| LPA (cm) | -0.017 +/- 0.63 | 0.95 | -0.050 +/- 0.44 | 0.68 |
| Upper ABR | -0.015 +/- 0.17 | 0.84 | -0.025 +/- 0.16 | 0.57 |
| Lower ABR | -0.039 +/- 0.23 | 0.70 | -0.11 +/- 0.22 | 0.09 |
| Delta ABR | -0.024 +/- 0.16 | 0.73 | -0.083 +/- 0.19 | 0.14 |

Mean values are provided with standard deviations

MPA denotes main pulmonary artery; RPA denotes right pulmonary artery; LPA denotes left pulmonary artery; ABR denotes artery-bronchus ratio; HPS denotes hepatopulmonary syndrome

*Delta ABR was calculated by subtracting the upper ABR from the lower ABR
