# Supplementary material for: CT Scan Does Not Differentiate Patients with Hepatopulmonary Syndrome from Other Patients with Liver Disease
Source: PLoS One. 2016 Jul 6;11(7):e0158637. doi: 10.1371/journal.pone.0158637 (PMC4934684; doi:10.1371/journal.pone.0158637)
Supplement: S3 Table — (DOCX) [file pone.0158637.s003.docx]

Supporting Information

**S3 Table. Comparison of pulmonary bronchovascular measurements among subdivided liver disease groups.**

|  | HPS^∞^ (n=23) | Subclinical HPS^∞^ (n=22) | Liver Dysfunction Only^∞^ (n=7) | Disease Group Comparisons (p-Value) |
| --- | --- | --- | --- | --- |
| MPA (cm) | 2.62 +/- 0.35 | 2.65 +/- 0.35 | 2.59 +/- 0.36 | 0.92 |
| RPA (cm) | 2.28 +/- 0.36 | 2.20 +/- 0.27 | 2.27 +/- 0.54 | 0.72 |
| LPA (cm) | 2.15 +/- 0.33 | 2.21 +/- 0.21 | 2.01 +/- 0.29 | 0.27 |
| Upper ABR | 1.08 +/- 0.13 | 1.08 +/- 0.12 | 1.04 +/- 0.11 | 0.71 |
| Lower ABR | 1.16 +/- 0.18 | 1.24 +/- 0.21 | 1.15 +/- 0.12 | 0.30 |
| Delta ABR* | 0.08 +/- 0.16 | 0.16 +/- 0.18 | 0.11 +/- 0.15 | 0.27 |

Mean values are provided with standard deviations

MPA denotes main pulmonary artery; RPA denotes right pulmonary artery; LPA denotes left pulmonary artery; ABR denotes artery-bronchus ratio; HPS denotes hepatopulmonary syndrome

^∞^Groups defined by contrast echocardiogram status

*Delta ABR was calculated by subtracting the upper ABR from the lower ABR
